# Mortality and recovery following moderate and severe acute malnutrition in children aged 6–18 months in rural Jharkhand and Odisha, eastern India: A cohort study

**DOI:** 10.1371/journal.pmed.1002934

**Published:** 2019-10-15

**Authors:** Audrey Prost, Nirmala Nair, Andrew Copas, Hemanta Pradhan, Naomi Saville, Prasanta Tripathy, Rajkumar Gope, Shibanand Rath, Suchitra Rath, Jolene Skordis, Sanghita Bhattacharyya, Anthony Costello, Harshpal S. Sachdev

**Affiliations:** 1 University College London, Institute for Global Health, London, United Kingdom; 2 Ekjut, Chakradharpur, Jharkhand, India; 3 Public Health Foundation of India, New Delhi, India; 4 Sitaram Bhartia Institute of Science and Research, New Delhi, India; The Hospital for Sick Children, CANADA

## Abstract

**Background:**

Recent data suggest that case fatality from severe acute malnutrition (SAM) in India may be lower than the 10%–20% estimated by the World Health Organization (WHO). A contemporary quantification of mortality and recovery from acute malnutrition in Indian community settings is essential to inform policy regarding the benefits of scaling up prevention and treatment programmes.

**Methods and findings:**

We conducted a cohort study using data collected during a recently completed cluster-randomised controlled trial in 120 geographical clusters with a total population of 121,531 in rural Jharkhand and Odisha, eastern India. Children born between October 1, 2013, and February 10, 2015, and alive at 6 months of age were followed up at 9, 12, and 18 months. We measured the children’s anthropometry and asked caregivers whether children had been referred to services for malnutrition in the past 3 months. We determined the incidence and prevalence of moderate acute malnutrition (MAM) and SAM, as well as mortality and recovery at each follow-up. We then used Cox-proportional models to estimate mortality hazard ratios (HRs) for MAM and SAM. In total, 2,869 children were eligible for follow-up at 6 months of age. We knew the vital status of 93% of children (2,669/2,869) at 18 months. There were 2,704 children-years of follow-up time. The incidence of MAM by weight-for-length z score (WLZ) and/or mid-upper arm circumference (MUAC) was 406 (1,098/2,704) per 1,000 children-years. The incidence of SAM by WLZ, MUAC, or oedema was 190 (513/2,704) per 1,000 children-years. There were 36 deaths: 12 among children with MAM and six among children with SAM. Case fatality rates were 1.1% (12/1,098) for MAM and 1.2% (6/513) for SAM. In total, 99% of all children with SAM at 6 months of age (227/230) were alive 3 months later, 40% (92/230) were still SAM, and 18% (41/230) had recovered (WLZ ≥ −2 standard deviation [SD]; MUAC ≥ 12.5; no oedema). The adjusted HRs using all anthropometric indicators were 1.43 (95% CI 0.53–3.87, *p* = 0.480) for MAM and 2.56 (95% CI 0.99–6.70, *p* = 0.052) for SAM. Both WLZ < −3 and MUAC ≥ 11.5 and < 12.5 were associated with increased mortality risk (HR: 3.33, 95% CI 1.23–8.99, *p* = 0.018 and HR: 3.87, 95% CI 1.63–9.18, *p* = 0.002, respectively). A key limitation of our analysis was missing WLZ or MUAC data at all time points for 2.5% of children, including for two of the 36 children who died.

**Conclusions:**

In rural eastern India, the incidence of acute malnutrition among children older than 6 months was high, but case fatality following SAM was 1.2%, much lower than the 10%–20% estimated by WHO. Case fatality rates below 6% have now been recorded in three other Indian studies. Community treatment using ready-to-use therapeutic food may not avert a substantial number of SAM-related deaths in children aged over 6 months, as mortality in this group is lower than expected. Our findings strengthen the case for prioritising prevention through known health, nutrition, and multisectoral interventions in the first 1,000 days of life, while ensuring access to treatment when prevention fails.

## Abstract 2

Audrey Prost and colleagues present a survey assessing levels of severe acute malnutrition in young children in 2 regions of rural India; they show levels are below suggested figures from the WHO.

## Author summary

**Why was this study done?:**

- Moderate acute malnutrition (MAM) and severe acute malnutrition (SAM) increase a child’s risk of dying. The World Health Organization (WHO) estimates that 10%–20% of children die following SAM, but this range comes from studies conducted over 15 years ago, mostly in African nations, and does not discriminate between children with and without medical complications.
- Recent studies from India have suggested that acute malnutrition may carry a lower risk of mortality than estimated by WHO, with substantial implications for the number of lives that could be saved by treating children as outpatients with ready-to-use therapeutic food. Unfortunately, most Indian studies conducted to date have been cross-sectional, small, or did not examine mortality risk with mid-upper arm circumference (MUAC), a commonly used anthropometric indicator to identify children with MAM or SAM.

**What did the researchers do and find?:**

- We conducted a cohort study as a secondary data analysis nested within a recently completed cluster-randomised controlled trial in 120 geographical clusters of Jharkhand and Odisha, two states of eastern India with a high prevalence of child undernutrition.
- We determined the incidence and prevalence of MAM and SAM, mortality, and recovery among 2,869 children followed up from 6 to 18 months of age.
- We found that SAM carried a lower case fatality rate (1.2%) than expected from WHO estimates (10%–20%), echoing results from three other Indian studies, which found case fatality rates ranging from 2.7% to 5.2% among children older than 6 months. We also found that over 40% of children with MAM and SAM remained acutely malnourished for over 3 months.

**What do these findings mean?:**

- Our data add to the accruing evidence that, in India, high rates of acute malnutrition in children over 6 months old are accompanied by lower case fatality rates than those reported in older, largely African studies.
- Overall, our findings suggest a need to prioritise the prevention of undernutrition by supporting known health, nutrition, and multisectoral interventions in the first 1,000 days of life.

## Introduction

In 2017, around 50 million children under 5 years globally were acutely malnourished [1]. Severe acute malnutrition (SAM) is defined as weight-for-height z score (WHZ)/weight-for-length z score (WLZ) < −3 standard deviation (SD) of the World Health Organization (WHO) 2006 Growth Standards, or mid-upper arm circumference (MUAC) < 11.5 cm [2]. Moderate acute malnutrition (MAM) is defined as WHZ/WLZ ≥ −2 and < −3 or MUAC ≥ 11.5 cm and < 12.5 cm [2]. Both MAM and SAM increase children’s risk of dying: a pooled analysis of 10 longitudinal studies from Asia, Africa, and South America found that children with moderate and severe wasting had 3- and 9-fold increased mortality rates, respectively, compared with children with WHZ > −1 [3]. Drawing on data collected mostly from hospitalised children before the advent of community-based management of acute malnutrition (CMAM), WHO estimated that between 10% and 20% of children with SAM generally die within 2–3 months without treatment, without discriminating between children with and without medical complications [4–6].

Nearly 18% of all children under 5 years globally live in India (121 out of 679 million), as do half of all children under 5 years affected by wasting (around 25.5 million out of 50.5 million) [1,7]. Indian policy makers, scholars, and activists relentlessly call for increased public funding to support the health, nutrition, sanitation, and social protection interventions that could reduce undernutrition [8–10]. Despite these efforts, scaling up preventive supplementary nutrition and detecting children with acute malnutrition has proved challenging. The Integrated Child Development Services’ (ICDS) *Anganwadi* (nutrition) workers should normally give supplementary food to pregnant and breastfeeding women, children under 3 years, and adolescent girls. They should also measure children’s WHZ monthly and refer severely wasted children to malnutrition treatment centres (MTCs) [10]. In 2014, however, a nationally representative survey found that only 21% of children aged 6–35 months received supplementary food at least 21 days per month [11]. When children with SAM were found and referred to MTC, families often feared the length and cost of hospital care [12]. There are currently no national-level data on the proportion of children with SAM who are identified and treated and few data on mortality following acute malnutrition in the community [9].

Using the Lives Saved Tool (LiST), the 2013 Lancet Commission on Maternal and Child Undernutrition estimated that scaling up outpatient feeding with ready-to-use therapeutic foods (RUTFs) for MAM and SAM at 90% coverage could save an estimated 435,210 children under 5 years every year [13,14]. Previous studies have suggested that mortality from acute malnutrition may be lower in India than in other settings [15–17]. Measuring actual mortality rates from MAM and SAM for children in India is critical to understand how many deaths could be prevented by scaling up treatment.

In this analysis, we estimated the prevalence and incidence of MAM and SAM, referrals, mortality, and recovery in a cohort of children aged 6–18 months in Jharkhand and Odisha, two Indian states with high levels of undernutrition, in the context of ordinary detection and treatment protocols from the ICDS, and with no ongoing CMAM programme.

## Methods

### Design

We conducted a cohort study as a secondary data analysis nested within a cluster-randomised controlled trial conducted between 2013 and 2016. The original trial tested a community intervention to improve linear growth among children under 2 years in rural eastern India through a participatory learning and action cycle of meetings with women’s groups and monthly visits for all mothers from the third trimester of pregnancy until a child’s second birthday. The trial was registered as ISCRTN 51505201 and Clinical Trials Registry of India number 2014/06/004664 [18]. This cohort study is reported in accordance with the Strengthening the Reporting of Observational Studies in Epidemiology (STROBE) guideline (S1 STROBE Checklist).

### Setting

The trial from which our cohort data are drawn took place in two districts: West Singhbhum, in Jharkhand, and Kendujhar, in Odisha. Both districts had high levels of child undernutrition: in 2015–2016, 38% of children under 5 years in West Singhbhum were wasted (13.5% severely wasted), as were 19% of children in Kendujhar (5% severely wasted) [7]. Over 75% of the population in the trial study areas belonged to *Adivasi* communities (Hindi: original inhabitants—i.e., indigenous), also known as Scheduled Tribes. Most families were engaged in agricultural labour, and 46% of women could read [18]. In total, 61% of households had electricity, and 1% had an improved toilet [18]. ICDS had unequal coverage in the study areas: we witnessed multiple interruptions in the provision of iron and folic acid, deworming tablets, and supplementary nutrition between 2013 and 2016. In the study clusters, village-based *Anganwadi* workers weighed children whose mothers attended monthly Village Health and Nutrition Days, offered supplementary nutrition to moderately underweight children, and referred those who were severely underweight to the MTC. Three treatment centres served the study communities: two in West Singhbhum and one in Kendujhar. They admitted children with WHZ < −3 SD or MUAC < 11.5 cm for inpatient treatment.

### Participants

In the trial that provided data for this study, 120 clusters were purposively created to approximate *Anganwadi* worker catchment areas of 1,000 people each [19]. Community-based informants identified all women in the third trimester of pregnancy residing in these clusters and invited them to participate in the study. All women who agreed were visited again within 72 hours of the birth, as well as 3, 6, 9, 12, and 18 months after the birth. For this cohort study, eligible participants were all singleton children born to pregnant women recruited between October 1, 2013, and February 10, 2015 (the trial recruitment period), and who were alive at 6 months of age. Data collectors made a minimum of three attempts to find children at each follow-up. We excluded data from infants younger than 6 months for three reasons. First, these infants are not currently targeted by community-based detection, referral, and treatment programmes for acute malnutrition in India. Second, other studies on mortality and recovery following MAM and SAM in India included children older than 6 months, and we wanted our analyses to be comparable with these. Finally, we had substantial missing data for WLZ or MUAC within 72 hours of birth (39.6%, or 1,188/3,001) because many mothers and infants were still in hospital or away from their homes; these missing data made it challenging to explore the association between birth anthropometry and mortality.

### Variables

We defined an episode of SAM as being newly identified with WLZ < −3, MUAC < 11.5 cm, and/or bilateral pitting oedema at 6, 9, or 12 months after not being SAM or missing at the previous follow-up. Similarly, we defined an episode of MAM as being newly identified with WLZ between −3 and −2 and/or MUAC between 11.5 and 12.5 cm at 6, 9, or 12 months after not being MAM or missing at the previous follow-up. Children could be acutely malnourished at two or more consecutive follow-ups; we reported such cases as a single episode of acute malnutrition. For example, a child found with SAM at both 6 and 9 months was counted as having a single episode of SAM. A child with SAM at 6 months who recovered at 9 months but was SAM again at 12 months was counted as having two episodes. Recovery was defined as WLZ ≥ 2 SD, MUAC ≥ 12.5, and no oedema. We considered a child to have died from acute malnutrition if they were acutely malnourished at the last follow-up before death (e.g., if a child was acutely malnourished at 6 months and died before the next follow-up at 9 months). If a child was SAM at 6 months, recovered at 9 months, and died at 10 months, the child was not considered to have died from SAM. The case fatality rate was calculated from among incident cases of MAM/SAM only.

### Measurement

Data collectors received 5 days of training on anthropometry, followed by 7 days of field practice. They measured children’s length using Shorr boards with a precision of 0.1 cm, their weight using Tanita BD-590 scales with 10-g graduations, and MUAC using UNICEF tapes with a precision of 0.1 cm. Data collectors also took part in three technical error of measurement (TEM) exercises: two before and one during the trial. In the first TEM, reliability coefficient values (R) were consistently over 0.95 for weight but below 0.5 for length and MUAC, which led to retraining and two further TEM exercises for length and MUAC. In the second and third TEMs, the average R was 0.98 for length and 0.78 for MUAC [20]. The third TEM took place in November 2014—i.e., nearly halfway through data collection. At each follow-up, data collectors checked whether the child was alive and asked whether they had ever been referred to an *Anganwadi* worker or MTC since the last visit. In total, 11% of interviews and measurements were conducted with a supervisor present. We had information on whether the children were alive at the last visit before migration. Data collectors sought to find each mother at least three times at each follow-up. Data collectors referred all children with WLZ < −3, oedema, or MUAC < 11.5 cm to local *Anganwadi* workers. This method was chosen to ensure that we did not create a parallel system for referrals to onwards facility-based treatment in MTC or bypass supplementary nutrition programmes.

### Statistical methods

We had no formal written analysis plan but took key decisions and drafted dummy tables prior to the analysis. All children recruited in the original trial were included in the analysis. We described the mothers’ and children’s socioeconomic characteristics and then examined differences between children retained in the cohort and those lost to follow-up using chi-squared tests for categorical variables and *t* tests for continuous variables. We used the zscore06 macro in Stata 14 to generate z scores of 2006 WHO Growth Standards and excluded WLZ values < −5 and > 5 as implausible, as recommended in WHO guidance [21]. We checked and flagged any cases in which height and weight did not increase over an interval of 3 months. We then calculated the prevalence and incidence of MAM and SAM at 6, 9, 12, and 18 months. Within each time period (6–9 months, 9–12 months, 12–18 months), we checked whether surviving children remained SAM, became MAM, or recovered from acute malnutrition at the next follow-up.

We used Cox-proportional models to estimate minimally and fully adjusted hazard ratios (HRs) with 95% CIs for mortality following MAM and SAM by WLZ, MUAC, and oedema (as relevant) combined and then for individual indicators [22]. The variables indicating MAM and SAM were time varying and updated at each follow-up time to reflect their impact on the risk of death only during an episode. In this analysis, we included the effect of prevalent MAM and SAM. We used multiple imputation by chained equations to account for missing measurements of WLZ or MUAC for children who had measurements from at least one follow-up. Thirty imputations sets were created, with missing values imputed conditional on values for the same variable at other times and also on whether the child subsequently died and the time of death or censoring. In all Cox-proportional models, we used robust standard errors to account for correlation within clusters (villages). We included data from both the intervention and control arms of the trial to maximise power and present HRs both adjusted and unadjusted for trial allocation. The intervention reduced infant mortality (adjusted odds ratio [aOR]: 0.63; 95% CI 0.39–1.00, *p* = 0.05) and underweight at 18 months (aOR: 0.81; 95% CI 0.66–0.99, *p* = 0.04) but did not reduce wasting (aOR: 0.88; 95% CI 0.71–1.08, *p* = 0.22) or increase mean MUAC at 18 months (adjusted difference: 0.05; 95% CI −0.08 to 0.186, *p* = 0.44). Adjusted analyses included variables for child sex, trial allocation arm, and district. A minimal data set and STATA DO file are included to enable readers to replicate our analyses (S1 Data and S1 DO File).

### Ethical considerations

We sought women’s individual informed consent in writing or by thumbprint during the enrolment interview in pregnancy and verbally before all subsequent interviews. The trial was reviewed and approved by the research ethics committee of the Public Health Foundation of India (June 2013, TRC-IEC-163/13), an independent ethics committee linked to Ekjut (May 2013, reference IEC/EKJUT/01), and University College London’s Research Ethics Committee (June 2013, reference 1881/002). The independent ethics committee linked to Ekjut approved this secondary data analysis after the completion of the trial.

## Results

### Retention and participants’ characteristics

**Fig 1:**
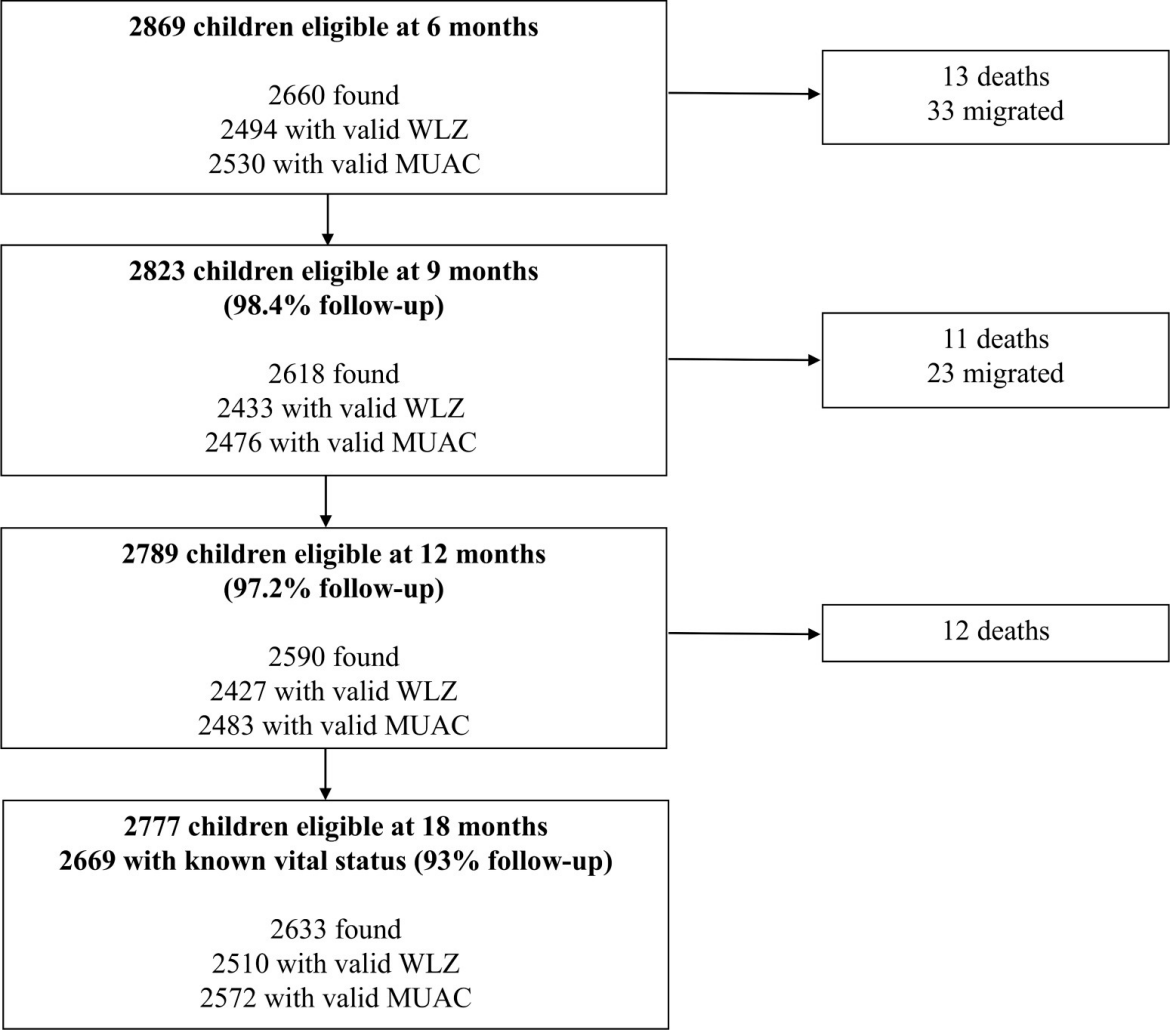
Flowchart describing the retention of children in the study. MUAC, mid-upper arm circumference; WLZ, weight-for-length z score.

Fig 1 shows the retention of participants in the study. In total, 2,869 singleton infants were eligible at 6 months, of which 36 died between 6 and 18 months, and 56 migrated permanently. We found 93% (2,669/2,869) of all children eligible at 6 months.

Table 1 describes mothers’ and children’s characteristics by follow-up status. Around half of infants were born in Kendujhar District in Odisha, and the other half were born in West Singhbhum, Jharkhand. Over two-thirds were born in *Adivasi* families. In total, 50.5% of infants were female, and 49.5% were male. Mothers’ mean height was 1.50 m (SD: 0.07), and children’s mean birth weight was 2.57 kg (SD: 0.4). We compared the characteristics of children with known vital status at 18 months with those of children lost to follow-up at 18 months. Children lost to follow-up were more likely to be from Jharkhand (72% of children lost to follow-up versus 48% of children with known status), female (59% of children lost to follow-up versus 50% of children with known status), and from poorer households (median multidimensional poverty score: −0.90 interquartile range [IQR]: −1.69, 0.63 for children lost to follow-up versus −0.56, IQR: −1.64, 1.39 for children with known status at 18 months).

**Table 1:** Characteristics of participants.

Characteristics	Children eligible at 6 months	Children with known status at 18 months^a^	Children lost to follow-up at 18 months^b^
***N***	2,869	2,669	200
**District (state), *n* (%)**			
West Singhbhum (Jharkhand)	1,425 (49.7)	1,281 (48.0)	144 (72.0)
Kendujhar (Odisha)	1,444 (50.3)	1,388 (52.0)	56 (28.0)
**Class or caste status, *n* (%)**			
Scheduled Tribe	2,192 (76.4)	2,033 (76.2)	159 (79.5)
Scheduled Caste	188 (6.5)	178 (6.7)	10 (5.0)
Other Backward Class	484 (16.9)	453 (17.0)	31 (15.5)
Other	5 (0.2)	5 (0.2)	0 (0)
**Multidimensional poverty score**^**c**^**, median (interquartile range)**	−0.63 (−1.64,1.23)	−0.56 (−1.64,1.39)	−0.90 (−1.69, 0.63)
**Mother’s age, mean (SD)**	24.0 (4.6)	24.0 (4.6)	23.7 (4.5)
**Maternal height in m, mean (SD)**	1.50 (0.07)	1.50 (0.07)	1.50 (0.07)
**Parity, mean (SD)**	2.4 (1.6)	2.4 (1.6)	2.5 (1.5)
**Child sex**			
Female	1,450 (50.5)	1,332 (49.9)	118 (59.0)
Male	1,419 (49.5)	1,337 (50.1)	82 (41.0)

^a^Children with known status at 18 months include those found alive at 18 months and those who died during follow-up.

^b^Lost to follow-up is defined as not found at 18 months and not dead.

^c^The Multidimensional Poverty Index comprised data on household assets, roof and floor materials, any deaths to a child under 5 years in the household, and any school-age children out of school, following the methodology described by Alkire and colleagues, which involves principal component analysis [22].

Abbreviation: SD, standard deviation

### Case fatality and mortality incidence following MAM and SAM

Children had 1,098 episodes of MAM beginning between 6 and 18 months and a MAM incidence rate of 406 per 1,000 children-years (1,098/2,704 children-years). In all, 12 children died during an incident MAM episode, five within 6 months of the start of the episode, and seven after 6 months. The case fatality from MAM was 0.4% (5/1,098) within 6 months and 1.1% (12/1,098) overall.

Children had 513 episodes of SAM beginning between 6 and 18 months and a SAM incidence rate of 190 per 1,000 children-years (513/2,704 children-years). In total, six children died during an incident SAM episode, four within 6 months of the start of the episode, and two after 6 months. The case fatality for SAM was 0.8% (4/513) within 6 months and 1.2% overall (6/513).

Table 2 shows the mortality incidence rates for MAM and SAM, as well as unadjusted and adjusted associations between MAM and SAM incidence and short-term (i.e., within the episode) mortality. A total of 68 children could not be included in analyses with multiple imputation: 10 had no WLZ measurement at any follow-up, 55 had no MUAC measurement at any follow-up, and three were immediately censored at the 6 months’ visit. S1 Table shows the number of missing values for each WLZ, MUAC, and oedema variable. The mortality incidence rate for MAM was 19.1 per 1,000 children-years. The mortality incidence rate for SAM was 20.1 per 1,000 children-years. The adjusted HR for MAM using all anthropometric indicators was 1.43 (95% CI 0.53–3.87, *p* = 0.480) and 2.56 (95% CI 0.99–6.70, *p* = 0.052) for SAM (overall *p* = 0.073). Lower MUAC, lower WLZ, and the presence of oedema were associated with increased risk of mortality (*p* = 0.003, *p* = 0.060, and *p* = 0.059, respectively).

**Table 2:** Associations between anthropometric indicators of MAM or SAM and short-term^a^ mortality.

Anthropometric indicators	Mortality incidence per 1,000 children-years	Minimally adjusted hazard ratios (95% CI), by category^b^	*p*	Minimally adjusted hazard ratios (95% CI), overall^b^	*p*	Fully adjusted hazard ratios (95% CI) by category^c^	*p*	Fully adjusted hazard ratios (95% CI), overall^c^	*p*
**All indicators**									
No acute	9.2	1				1			
MAM	19.1	1.40 (0.52–3.77)	0.508			1.43 (0.53–3.87)	0.480		
SAM	19.7	2.23 (0.80–6.19)	0.124	1.47 (0.89–2.43)	0.128	2.56 (0.99–6.70)	0.052	1.57 (0.96–2.55)	0.070
**WLZ**									
≥−2	9.3	1				1			
≥−3 and <−2	21.1	1.14 (0.46–2.83)	0.773			1.20 (0.47–3.04)	0.702		
<−3	25.0	2.80 (0.94–8.37)	0.064	1.53 (0.88–2.65)	0.131	3.33 (1.23–8.99)	0.018	1.65 (0.98–2.78)	0.061
**MUAC**									
≥12.5	10.3	1				1			
≥11.5 and <12.5	24.4	3.81 (1.57–9.24)	0.003			3.87 (1.63–9.18)	0.002		
<11.5	11.3	1.95 (0.24–15.5)	0.527	2.13 (1.25–3.62)	0.005	1.89 (0.23–15.2)	0.551	2.10 (1.25–3.53)	0.005
**Oedema**									
No	12.2	1				1			
Yes	67.4	4.79 (0.61–37.6)	0.136			5.87 (0.94–36.5)	0.058		

^a^Hazard ratios reflect the impact of a malnutrition indicator until its value is updated at a subsequent follow-up visit.

^b^Adjusted for clustering using robust standard errors.

^c^Adjusted for child’s sex, trial allocation, district, and clustering using robust standard errors.

Abbreviations: MAM, moderate acute malnutrition; MUAC, mid-upper arm circumference; SAM, severe acute malnutrition; WLZ, weight-for-length z score

### Nutritional status among survivors

S2 Table describes the prevalence of MAM and SAM at the start of each follow-up period (i.e., at 6, 9, and 12 months), along with the vital and nutritional status of children at the next follow-up. In total, 99% of all children with SAM at 6 months (227/230) were alive after 3 months, 40% (92/230) were still SAM, and 18% (41/230) had ‘recovered’ (WLZ ≥ −2 SD; MUAC ≥ 12.5; no oedema). Only 5% (32/584) of children with SAM at 6, 9, or 12 months were ever referred to an MTC. The coverage of preventive nutrition services was also unequal: for example, 66% (1,917/2,869) of children aged 6 months were weighed by an *Anganwadi* worker, but only 13% (382/2,869) had mothers who received nutrition counselling.

### Outcomes up to 12 months after MAM and SAM

**Fig 2:**
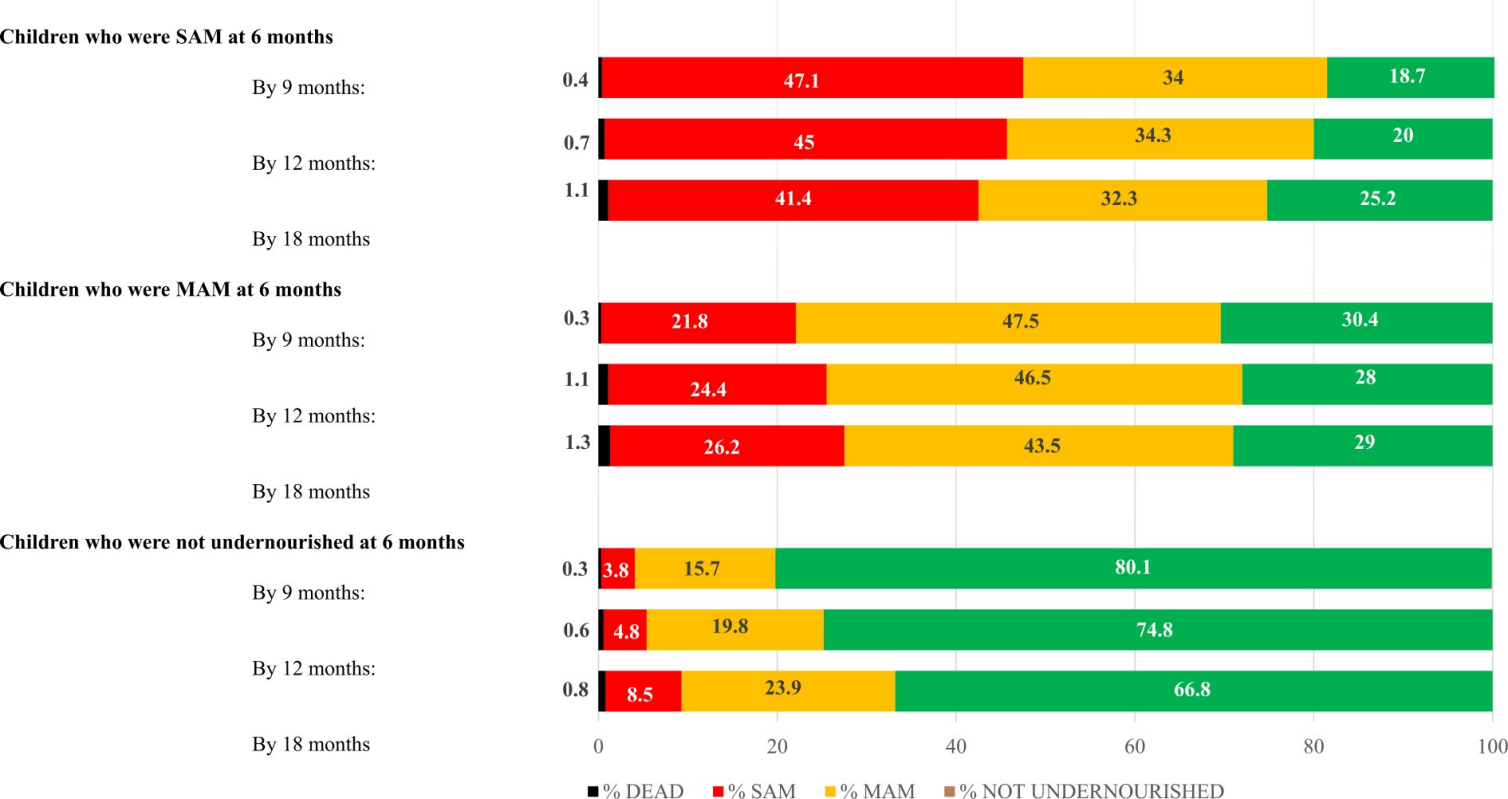
Long-term outcomes for children with and without acute malnutrition at 6 months, using a data set with multiple imputation. MAM, moderate acute malnutrition; SAM, severe acute malnutrition.

Fig 2 shows outcomes at 9, 12, and 18 months for children who were not undernourished or had MAM or SAM at 6 months. These were derived after multiple imputation for missing WLZ, MUAC, and oedema data. The proportions of children with MAM or SAM at 6 months who had died by 18 months were 1.3% and 1.1%, respectively. These were not very different, in absolute terms, to the proportion of non-acutely malnourished children who died (0.8%). Recovery from MAM and SAM was relatively low and stable over time. Recovery out of acute undernutrition was similar for MAM or SAM, particularly by 18 months.

## Discussion

We found a high incidence of MAM and SAM among rural, largely tribal communities of Jharkhand and Odisha. A substantial proportion (>40%) of children with MAM and SAM remained acutely malnourished for over 3 months, and 99% of children with SAM at 6 months survived for 3 months or more, suggesting that, in this context, MAM and SAM are persistent. WLZ < −3 was associated with increased mortality risk, as expected, but MUAC ≥ 11.5 and < 12.5 was associated with a greater mortality risk than MUAC < 11.5. The finding that children with MUAC ≥ 11.5 and < 12.5 had a higher mortality risk is counterintuitive and deserves further investigation. *Anganwadi* workers referred only 5% of SAM cases to MTC, highlighting a considerable treatment gap.

Our most salient finding is that SAM carried a much lower case fatality rate (1.2%) than expected from older, largely African studies and WHO estimates (10%–20%). If the two deceased children for whom we did not have any anthropometry data had died following SAM, the case fatality rate would have been 1.5% (8/513), still much below 10%–20%.

Three other Indian studies with different designs but broadly similar inclusion criteria offer data for comparison. The first was a follow-up study of children initially screened as having an MUAC < 11.5 cm as part of a multicentric trial in poor urban and rural communities in New Delhi, Udaipur, and Vellore [23]. Case fatality among children aged 6–23 months with MUAC < 11.5 cm was 4.3% (6/140) after 12 months [24]. A second study conducted in rural Uttar Pradesh found a case fatality of 2.7% among 409 children aged 6 months to 5 years followed up between 0.6 and 17.8 months after severe wasting [25]. In a third study from Bihar, Burza and colleagues followed up defaulters from a WHO-standard RUTF programme who had MUAC < 11.5 cm [26]. Case fatality among these defaulters—who tended to be younger children and girls—was 5.2% within 18 months (36/692). All of these case fatality rates, with varying durations of follow-up, are below WHO estimates (10%–20%) and closer to the 4% (range: 2%–7%) case fatality rate that a 2013 expert panel considered normal in the context of a CMAM programme [27].

There are at least four possible explanations for the considerable difference between case fatality rates in these recent Indian studies and the 10%–20% estimates drawn from older, largely African studies. First, the studies upon which WHO estimates are based included mostly hospitalised children who were more likely to have SAM with medical complications and, therefore, higher mortality rates [6].

A second possible explanation is that the most vulnerable children in our rural study areas died before reaching 6 months. In the trial’s control clusters, the infant mortality rate was 64 per 1,000 live births; 64% (116/181) of all infant deaths across both trial areas occurred in the first month of life, and 86% (157/181) occurred in the first 6 months [18]. In 2015, the prevalence of low birth weight per 100 births was greater in southern Asia than in sub-Saharan Africa (26.4%; uncertainty range [UR]: 18.6–35.2 versus 14.0%, UR: 12.2–17.2) [28]. Around half of neonatal deaths in India (370,000 of 698,000 neonatal deaths in 2015) are caused by prematurity or low birth weight, and these factors also accounted for 46.1% of all under-5 deaths in 2017 (95% uncertainty interval: 44.5–47.6) [29,30]. In rural India, undersize is probably taking its heaviest toll in the first 6 months of life, before treatment with RUTF becomes relevant.

A third source of variation in case fatality rates may lie in differences in infection burdens between South Asian and African settings. In 2017, the disability-adjusted life years rate for communicable maternal, neonatal, child, and nutritional diseases was 26,492 per 100,000 in sub-Saharan Africa, roughly double that in South Asia (13,197 per 100,000) [31]. HIV prevalence is 4.1% among 15–49-year-olds in sub-Saharan Africa versus 0.2% in South Asia, including India, and several studies have demonstrated that HIV infection interacts with undernutrition to increase the risk of death [31–33].

A final explanation may lie in the developmental origins of adult health and disease. Mothers in our study areas had an average height of 1.5 m [17]. During pregnancy, half of them had an MUAC < 23 cm, and one-third of them did not have diets with minimum diversity [17]. Around one-third (33%) of infants were low birth weight, and over 65% were stunted by 18 months [18]. These data reflect intergenerational, chronic undernutrition inflicted by poverty [34]. Indian infants often have a ‘thin-fat’ phenotype: they have small abdominal viscera and low muscle mass but preserve body fat in utero that can track into birth and young adulthood, depending on the nutritional environment [35]. Studies have found that body fat–related measurements such as skinfold thickness, abdominal circumference, and MUAC are often similar between Indian babies and Western infants, despite low birth weight [36].The hidden adiposity of Indian infants may give small infants a survival advantage by acting as a form of energy reserve available to maintain body temperature and brain development in times of nutritional deprivation, albeit with adverse consequences for noncommunicable disease risk in later life [37]. It is therefore possible that thinness in infancy and early childhood does not carry the same mortality risk in this setting as in others. The interplay between undernutrition, immune dysfunction, and mortality risk is complex, as summarised in recent reviews [38–40]. Integrating longitudinal immune assessments into ongoing trials of nutrition and water sanitation and hygiene (WASH) interventions will help elucidate when and how immune dysfunction and undernutrition interact and help better target public health interventions to lower mortality risk.

Our study had several strengths. To our knowledge, it is the largest Indian community-based longitudinal study of mortality following MAM and SAM in the absence of management of uncomplicated SAM with RUTF for children older than 6 months. The three TEM exercises undertaken by the data collection team enhance our trust in the accuracy of anthropometry data. We had information about vital status for 93% of infants eligible for follow-up at 6 months by the end of the study period.

Our study had three main limitations. We had missing anthropometry data for some children, including fully missing data for two of the 36 children who died. We attempted to address possible bias caused by missing data by using multiple imputation to provide more accurate estimates of mortality risk but were not able to use imputation for 68 children who had no WHZ or MUAC measurements at any time point. A second limitation is that we did not follow children up until 59 months of age. The study from Uttar Pradesh by Kapil and colleagues found a case fatality rate of 2.7% among children aged 6–60 months; this is higher than among the younger children in our study but still not within WHO’s estimated range [25]. Our results are therefore likely to be generalisable to young children aged 6–24 months in other rural, underserved Indian settings and may provide a modest underestimate of case fatality for children aged 6–59 months. Finally, monitoring and referral systems for SAM in the original trial differed from those available in the general population, and this may have contributed to lowering mortality. We have little evidence to support this hypothesis, however: infant mortality in the trial’s control area (63 per 1,000 live births) was similar to that reported in the 2011–2012 Annual Health Surveys for our two study districts (56 per 1,000 live births in Kendujhar and 58 in West Singhbhum), suggesting that the trial monitoring systems did not lead to large reductions in mortality in and of themselves [41].

If data from poor urban and rural communities in Delhi, Bihar, Uttar Pradesh and our own data from Jharkhand and Odisha are representative of the contemporary Indian situation, then case fatality rates following SAM in Indian children older than 6 months are substantially lower than those estimated from earlier studies conducted in African countries. In other Indian studies assessing progress following inpatient and/or community care for SAM, rates of recovery were often lower than recommended in SPHERE guidance, and relapse was common [42–44]. For example, in a recent follow-up study of 150 children discharged from MTCs in Jharkhand, 52% of children had relapsed into severe wasting 2 months after discharge [45]. In light of these findings, scaling up outpatient RUTF treatment for children over 6 months without concurrently strengthening ICDS, WASH, and the social protection interventions that support the intergenerational prevention of undernutrition may not have the large effects on mortality predicted by the 2013 Lancet Maternal and Child Nutrition Series in India and other South Asian contexts [17]. Prevention can work: a recent intervention combining crèches, participatory meetings with women’s groups, and home visits reduced wasting, underweight, and stunting among children under 3 years old in Jharkhand and Odisha [46]. A recent UNICEF-led consultation aptly called for a new approach to wasting in South Asia, one that positions prevention as the ‘first priority’ and ensures access to treatment when prevention fails [47]. Our findings support such a strategy.

In conclusion, we found a high incidence of acute malnutrition among children older than 6 months in rural eastern India but a lower than expected case fatality rate following SAM. This finding has been replicated in at least three other Indian studies. Given that the risk of mortality is lower than expected among children older than 6 months and that many deaths occur because of prematurity or low birth weight during the neonatal period, outpatient treatment for SAM using RUTF for children over 6 months may be too late to avert a substantial number of deaths from undernutrition in Indian children. This further strengthens the case for prioritising prevention through known health, nutrition, and multisectoral interventions in the first 1,000 days of life.

## Supporting information

S1 STROBE Checklist STROBE, Strengthening the Reporting of Observational Studies in Epidemiology. (DOCX)

S1 Data (CSV)

S1 DO File (DO)

S1 Table (DOCX)

S2 Table (DOCX)

## Abbreviations

aOR: adjusted odds ratio
CMAM: community-based management of acute malnutrition
HR: hazard ratio
ICDS: Integrated Child Development Services
IQR: interquartile range
LiST: Lives Saved Tool
MAM: moderate acute malnutrition
MTC: malnutrition treatment centre
MUAC: mid-upper arm circumference
RUTF: ready-to-use therapeutic food
SAM: severe acute malnutrition
SD: standard deviation
STROBE: Strengthening the Reporting of Observational Studies in Epidemiology
TEM: technical error of measurement
UR: uncertainty range
WASH: water sanitation and hygiene
WHO: World Health Organization
WHZ: weight-for-height z score
WLZ: weight-for-length z score
