# Supplementary material for: Mortality and recovery following moderate and severe acute malnutrition in children aged 6–18 months in rural Jharkhand and Odisha, eastern India: A cohort study
Source: PLoS Med. 2019 Oct 15;16(10):e1002934. doi: 10.1371/journal.pmed.1002934 (PMC6793843; doi:10.1371/journal.pmed.1002934)
Supplement: S1 Table — (DOCX) [file pmed.1002934.s004.docx]

### Supplementary Table 1: Missing values imputed

|  | 6 monthsN= 2869 * | 9 monthsN= 2836 * | 12 monthsN=2813 * | 18 monthsN=2777 * |
| --- | --- | --- | --- | --- |
| WLZ, n (%) | 296 (10.3) | 362 (12.8) | 368 (13.1) | 326 (11.7) |
| MUAC, n (%) | 277 (9.6) | 328 (11.6) | 322 (11.4) | 279 (10.0) |
| Oedema, n (%) | 337 (11.7) | 324 (11.4) | 324 (11.5) | 279 (10.0) |

### * Eligible children (i.e. alive and not migrated)
