# Supplementary material for: Mortality and recovery following moderate and severe acute malnutrition in children aged 6–18 months in rural Jharkhand and Odisha, eastern India: A cohort study
Source: PLoS Med. 2019 Oct 15;16(10):e1002934. doi: 10.1371/journal.pmed.1002934 (PMC6793843; doi:10.1371/journal.pmed.1002934)
Supplement: S2 Table — (DOCX) [file pmed.1002934.s005.docx]

### S2 Table: Mortality, survival and recovery following moderate and severe acute malnutrition from 6 to 18 months, using weight-for-length, MUAC and oedema

|  |  | **Vital status at next follow-up** | | | | | | **Nutritional status among survivors at next follow-up** | | | | | | | |
| --- | --- | --- | --- | --- | --- | --- | --- | --- | --- | --- | --- | --- | --- | --- | --- |
|  | **Prevalent cases**^a^  **N %** | **Missing**  **n %** | | **Died** ^b^  **n %** | | **Survived**  **n %** | | **No acute malnutrition**  **N %** | | **MAM** ^c^  **n %** | | **SAM** ^c^  **n %** | | **Missing**  **n %** | |
| 6-9 months |  |  |  |  |  |  |  |  |  |  |  |  |  |  |  |
| SAM | 230c 8.0 | 2 | 0.9 | 1 | 0.4 | 227 | 98.7 | 41 | 18.1 | 67 | 29.5 | 92 | 40.5 | 27 | 11.9 |
| MAM | 628c 21.9 | 10 | 1.6 | 3 | 0.5 | 615 | 97.9 | 172 | 28.0 | 267 | 43.4 | 118 | 19.2 | 58 | 9.4 |
| No acute malnutrition | 1665 58.0 | 18 | 1.1 | 6 | 0.4 | 1641 | 98.6 | 1218 | 74.2 | 223 | 13.6 | 54 | 3.3 | 146 | 8.9 |
| Not found or missing anthropometry | 346 12.1 | 8 | 2.3 | 2 | 0.6 | 337 | 97.1 | 128 | 38.0 | 63 | 18.7 | 30 | 8.9 | 116 | 34.4 |
| Total | 2869 100.0 | 37 | 1.3 | 12 | 0.4 | 2820 | 98.3 | 1559 | 55.3 | 620 | 22.0 | 294 | 10.4 | 347 | 12.3 |
| 9-12 months |  |  |  |  |  |  |  |  |  |  |  |  |  |  |  |
| SAM | 294 10.4 | 7 | 2.4 | 3 | 1.0 | 284 | 96.6 | 28 | 9.9 | 92 | 32.4 | 139 | 48.9 | 25 | 8.8 |
| MAM | 620 22.0 | 18 | 2.9 | 1 | 0.2 | 601 | 96.9 | 169 | 28.1 | 281 | 46.8 | 101 | 16.8 | 50 | 8.3 |
| No acute malnutrition | 1559 55.2 | 19 | 1.2 | 3 | 0.2 | 1537 | 98.6 | 1142 | 74.3 | 242 | 15.7 | 47 | 3.1 | 106 | 6.9 |
| Not found or missing anthropometry | 350 12.4 | 9 | 2.6 | 0 | 0.0 | 341 | 97.4 | 130 | 38.1 | 73 | 21.4 | 35 | 10.3 | 103 | 30.2 |
| Total | 2823 100.0 | 53 | 1.9 | 7 | 0.2 | 2763 | 97.9 | 1469 | 53.2 | 688 | 24.9 | 322 | 11.7 | 284 | 10.3 |
| 12-18 months |  |  |  |  |  |  |  |  |  |  |  |  |  |  |  |
| SAM | 322 11.5 | 17 | 5.3 | 0 | 0.0 | 305 | 94.7 | 46 | 15.1 | 114 | 37.4 | 137 | 44.9 | 8 | 2.6 |
| MAM | 688 24.7 | 30 | 4.4 | 1 | 0.1 | 657 | 95.5 | 186 | 28.3 | 330 | 50.2 | 127 | 19.3 | 14 | 2.1 |
| No acute malnutrition | 1469 52.7 | 72 | 4.9 | 3 | 0.2 | 1394 | 94.9 | 1076 | 77.2 | 245 | 17.6 | 44 | 3.2 | 29 | 2.1 |
| Not found or missing anthropometry | 310 11.1 | 29 | 9.4 | 4 | 1.3 | 277 | 89.4 | 141 | 50.9 | 95 | 34.3 | 27 | 9.7 | 14 | 5.1 |
| Total | 2789 100.0 | 148 | 5.3 | 8 | 0.3 | 2633 | 94.4 | 1449 | 55.0 | 784 | 29.8 | 335 | 12.7 | 65 | 2.5 |

^a^ A prevalent case of SAM is defined as WLZ<-3SD and/or MUAC<11.5cm and/or bilateral pitting oedema at the start of the follow-up period. A prevalent case of MAM is defined as WLZ ≥-3SD and <-2SD, and/or MUAC≥-11.5 cm and <12.5cm at the start of the follow-up period.

^b^ This table only includes deaths detected by the follow-up after an episode (n=27). A further nine deaths occurred after six months of initial detection, totalling 36 deaths.

^c^ There were 193 incident SAM episodes and 474 incident MAM episodes at six months.
